# Uncoupling protein 3 attenuates generation of reactive oxygen species by interacting with thioredoxin 2 in the mitochondrial intermembrane space

**DOI:** 10.1186/ar3625

**Published:** 2012-02-09

**Authors:** Katsuya Hirasaka, Edward M Mills, Shohei Kohno, Tomoki Abe, Chika Ikeda, Tasuku Maeda, Shigetada Kondo, Ayako Maita, Yuushi Okumura, Takeshi Nikawa

**Affiliations:** 1 Department of Nutritional Physiology, Institute of Health Biosciences, University of Tokushima, Tokushima, 770-8503, Japan; 2 Division of Pharmacology/Toxicology, University of Texas at Austin, Austin, TX 78712, USA

## 

Uncoupling protein 3 (UCP3) is primarily expressed in the inner membrane of skeletal muscle mitochondria. It has been proposed that UCP3 reduces production of reactive oxygen species (ROS) and oxidative damage. However, the mechanisms by which UCP3 attenuates ROS production are not well understood. Here we report that UCP3 interacts with the non-processed form of thioredoxin 2 (Trx2), a redox protein that is localized in mitochondria, but not processed Trx2, which is involved in cellular responses to ROS. The hydrophilic sequences within the N-terminal tail of UCP3, which faces the intermembrane space, are necessary for binding to Trx2. In addition, Trx2 directly associated with UCP3 through a mitochondrial targeting signaling sequence, was processed in the intermembrane space, and thereby allowing redox reactions. A bimolecular fluorescence complementation analysis demonstrated that the interaction of these proteins occurs in the mitochondrial intermembrane space. Furthermore, increased UCP3 expression significantly attenuated ROS production in isolated mitochondrial without effects on membrane potential, however this effect is lost by Trx2 knock down. These results suggest that UCP3 binds to Trx2 in the mitochondrial intermembrane space and attenuates ROS production.

